# Peer Review of “Use of a Specialist Telephone Consultation Line for Long COVID in Primary Care in British Columbia: Retrospective Descriptive Quality Improvement Study”

**DOI:** 10.2196/90935

**Published:** 2026-02-10

**Authors:** Anonymous

**Keywords:** internal medicine, long COVID, COVID-19, SARS-CoV-2, GP, general practice, general practitioner, consult, respiratory, infectious, respiration, primary care, telephone, telehealth

*This is the peer-review report for “Use of a Specialist Telephone Consultation Line for Long COVID in Primary Care in British Columbia: Retrospective Descriptive Quality Improvement Study.”*

## Round 1 Review

With all due respect to the authors for their work, I personally found the language of the article [1] to be fair, but there are the following problems:

1. The study design was relatively simple, with only age and gender collected for basic characteristics and no mention of past medical history, which had a greater impact on the study results, especially since the study results showed a high rate of reported respiratory symptoms. In addition, the 40‐49 year age group also had a high prevalence of chronic respiratory illnesses; previous respiratory illnesses are bound to worsen to varying degrees after a COVID infection. Despite the high probability of missing visits or ambiguous data, the collection of past medical history is something that I personally feel should have been added, and missing data need to be accounted for.
2. As a quality improvement study, I believe that the original COVID–general internal medicine–Post-Infection Care Rapid Access to Consultative Expertise line should be introduced (such as through flowcharts) to identify problems in the follow-up process and problems affecting the results of the study and to propose more specific improvement measures such as special training for follow-up personnel to guide the enrolled patients to more accurately provide the information needed for the study.
3. There are too many confounding factors affecting the results, and the author team does not seem to have mentioned measures to minimize the impact of confounding factors on the results of the study.
